# Identification of Prostate Cancer Risk Genetics Biomarkers Based on Intergraded Bioinformatics Analysis

**DOI:** 10.3389/fsurg.2022.856446

**Published:** 2022-03-17

**Authors:** Xiangdong Liang, Yanchao Wang, Long Pei, Xiaoliang Tan, Chunhui Dong

**Affiliations:** Department of Urology, The Fourth Hospital of Hebei Medical University, Shijiahzuang, China

**Keywords:** prostate cancer (PCa), bioinformatics analysis, immune cell infiltration, survival analysis, ARHGEF38, KCNK3, AK5

## Abstract

**Background:**

Prostate cancer (PCa) is one of the most popular cancer types in men. Nevertheless, the pathogenic mechanisms of PCa are poorly understood. Hence, we aimed to identify the potential genetic biomarker of PCa in the present study.

**Methods:**

High-throughput data set GSE46602 was obtained from the comprehensive gene expression database (GEO) for screening differentially expressed genes (DEGs). The common DEGs were further screened out using The Cancer Genome Atlas (TCGA) dataset. Functional enrichment analysis includes Gene Ontology (GO) and Kyoto Encyclopedia of Genes and Genomes (KEGG) to study related mechanisms. The Cox and Lasso regression analyses were carried out to compress the target genes and construct the high-risk and low-risk gene model. Survival analyses were performed based on the gene risk signature model. The CIBERSORT algorithm was performed to clarify the correlation of the high- and low-risk gene model in risk and infiltration of immune cells in PCa.

**Results:**

A total of 385 common DEGs were obtained. The results of functional enrichment analysis show that common DEGs play an important role in PCa. A three-gene signature model (*KCNK3, AK5*, and *ARHGEF38*) was established, and the model was significantly associated with cancer-related pathways, overall survival (OS), and tumor microenvironment (TME)-related immune cells in PCa.

**Conclusion:**

This new risk model may contribute to further investigation in the immune-related pathogenesis in progression of PCa.

## Introduction

Prostate cancer is regarded as the second foremost reason of death from cancer in men that affects men's health worldwide (1–3), especially in European and American countries (4–6). Surgery and radiotherapy are considered to be the most effective treatment strategies at an early stage (7). Androgen deprivation therapy (ADT) is the main treatment for advanced prostate cancer (8). Although the incidence rate of prostate cancer is low in China, the life span and the dietary structure change are also increasing year by year, with poor differentiation, high malignancy, and poor prognosis (9). Metastatic events are the main cause of death for men with prostate cancer for the reason that PCa can spread to multiple organs in the body (10). The 5 year survival ratio of metastatic prostate cancer is only about 30%. Nevertheless, androgen-deprivation therapy, in general, is not curative. Patients can develop to castration-resistant prostate cancer, which is lethal (10, 11). Therefore, timely diagnosis of PCa is of great importance for treatment and prognosis.

As a heterogeneous disease, the progression of PCa is closely associated with genome instability (12). Studies have indicated that PCa includes a complicated pathogenesis driven by multiple molecular pathways that are highly associated with the survival, metabolic, and metastatic characteristics of aggressive cancers (13). The genes are considered as loci of susceptibility to tumorigenesis in humans (14). Alterations in expression of genetic biomarker have been reported in various tumors (15, 16). However, in the current study, the development and progression of PCa are poorly understood at molecular and genetic levels.

Tumor immunotherapy is becoming a pillar of the cancer therapy armamentarium (17, 18). A growing number of studies suggest that immune responses may be involved in the clinical outcome of prostate cancer (19–21). As we know, tumor-infiltrating immune cells play a very important regulatory role in the tumor microenvironment and are an attractive therapeutic target (22). PCa has been shown to be significantly associated with immune infiltration in several clinical and genomic trials (23, 24). Multiple genes, such as *COL3A1, RAC1, FN1, SDC2*, and *TNB-585*, have been proved to be associated with high infiltration immune cells in prostate cancer (25, 26).

Bioinformatics analysis based on high-throughput next-generation sequencing technology enhances our understanding of gene expression function in cancer (27). In addition, transcriptomic data analysis is a useful method to identify DEGs at the genome-wide level, which is beneficial for our better understanding of the potential molecular mechanisms of the regulatory role of gene expression (28). Hence, the application of bioinformatics is useful for the investigation into the underlying mechanism of molecular cell biology in PCa.

In this study, through the integrated analysis of PCa data from the public databases, we screened out the potential genetic biomarkers that play a vital role in PCa. Functional enrichment analysis was performed to study the related underlying mechanism and signaling pathways, including gene ontology (GO) and Kyoto Encyclopedia of Genes and Genomes (KEGG). The Cox regression and Lasso regression analyses were conducted to construct the high- and low-risk gene model. The CIBERSORT algorithm was utilized to clarify the correlation of high-risk and low-risk gene model in risk and infiltration of immune cells in PCa. Integrated analysis for identifying novel biomarkers might be beneficial to PCa treatment and has a better understanding of the pathological mechanism.

## Materials and Methods

### Data Preparation and Processing

The high-throughput datasets GSE46602 (29) of PCa was acquired from the public GEO database (https://www.ncbi.nlm.nih.gov/geo). The GSE46602 dataset contains 36 tumor tissues and 14 normal prostate biopsies. In addition, the RNA-sequencing data of PCa and normal control tissues were obtained from the The Cancer Genome Atlas (TCGA) database (https://portal.gdc.cancer.gov/). The PCa samples were analyzed using integrated bioinformatics methods, and samples without complete clinical information were excluded. In addition, the RNA-seq data and clinical information of 492 PCa samples were obtained from the TCGA database.

### Identification of Differential Expression Genes (DEGs) in PCa

The RNA expression profile carried out normalization with the Affy package. The RNA expression profile was analyzed by the limma R package (30). The DEGs were displayed in the form of the volcano plot and heat maps and identified old change of log_2_ > 1.5 and *p*-value < 0.05. The R ggplot2 package in the R analysis platform was plotted to present the heat maps and clustering of DEGs.

### Functional Enrichment Analysis

To evaluate the potential role of common DEGs in PCa development, GO functional enrichment analyses were used to analyze the biological process (BP), cellular component (CC), and molecular function (MF) of DEGs. In the current study, GO analysis of DEGs was performed by DAVID (31, 32) (https://david.ncifcrf.gov/conversion.jsp). Functional enrichment analysis of KEGG was mainly used to analyze the signaling/metabolic pathway through which differentially expressed genes may perform their biological functions (33). *P*-value < 0.05 was considered as the critical value for screening the significant enrichment pathway.

### Cox Regression and Lasso Regression Analysis

Lasso regression was performed to characterize the high frequency features (34). Then, the univariate Cox regression analysis was performed to screen out the genes with significant correlation (*p* < 0.05). Next, the least absolute shrinkage and selection operator (lasso) regression was carried out to further reduce the number of genes. We used the glmnet3 package (35) to conduct Lasso cox regression analysis based on machine learning to identify the optimal prognostic signature. In the next step, multivariate regression analysis was carried out, step function was used for stepwise regression screening, and finally, the model constructed by the three genes was obtained. Based on the expression of 3 genes in the constructed model, a new risk scoring model was constructed by multivariate Cox regression evaluation. The risk score was then determined, and the sample was stratified into high- and low-risk groups according to the median risk score to verify whether the risk score was an independent predictor.

### Immune Infiltration by the Cibersort Analysis

CIBERSORT algorithm (36) was a mean to discriminate a signature between twenty-two human immune cell phenotypes, including memory B cells, activated CD4+ T cells, neutrophils, and so on. The CIBERSORT algorithm was used to quantify the proportion of immune cells in PCa. PCa gene expression profiles from the TCGA database were uploaded to the CIBERSORT, and 1,000 permutations were run. Data with *p*-value < 0.05 after the CIBERSORT were performed for the analysis to improve the accuracy of the deconvolution method. The CIBERSORT software package in R software was used for data analysis. Wilcox test was used to compare the relative abundance of the TIIC between high- and low-risk groups.

### Survival Analysis

Correlation between 3 genes and the overall survival of patients with PCa were analyzed utilizing the GEPIA database (37). According to each hub gene's best-separation cutoff value, samples of patients with PCa within the dataset were divided into two groups to obtain the Kaplan–Meier (K–M) survival curves. ROC curves were carried out to investigate the prognostic value in 1, 3, and 5 years by utilizing the survival ROC package (v1.0.34) based on the GSE46602 dataset.

### Gene Set Enrichment Analysis (GSEA) of High- and Low-Risk Patients With Pc

GSEA (Version 4.1.0) was used to screen for gene clusters associated with risk score phenotypes, which were overrepresented in large groups of genes. Enriched *P*-values were calculated based on 1,000 permutations; FDR values were calculated using the Benjamini–Hochberg multiple test correction program (*p* < 0.05). In addition, the enrichment pathways of each phenotype were classified by nominal *P*-value and standard enrichment score (NES) (38).

## Results

### Identification of Differentially Expressed Genes

**Figure 1:**
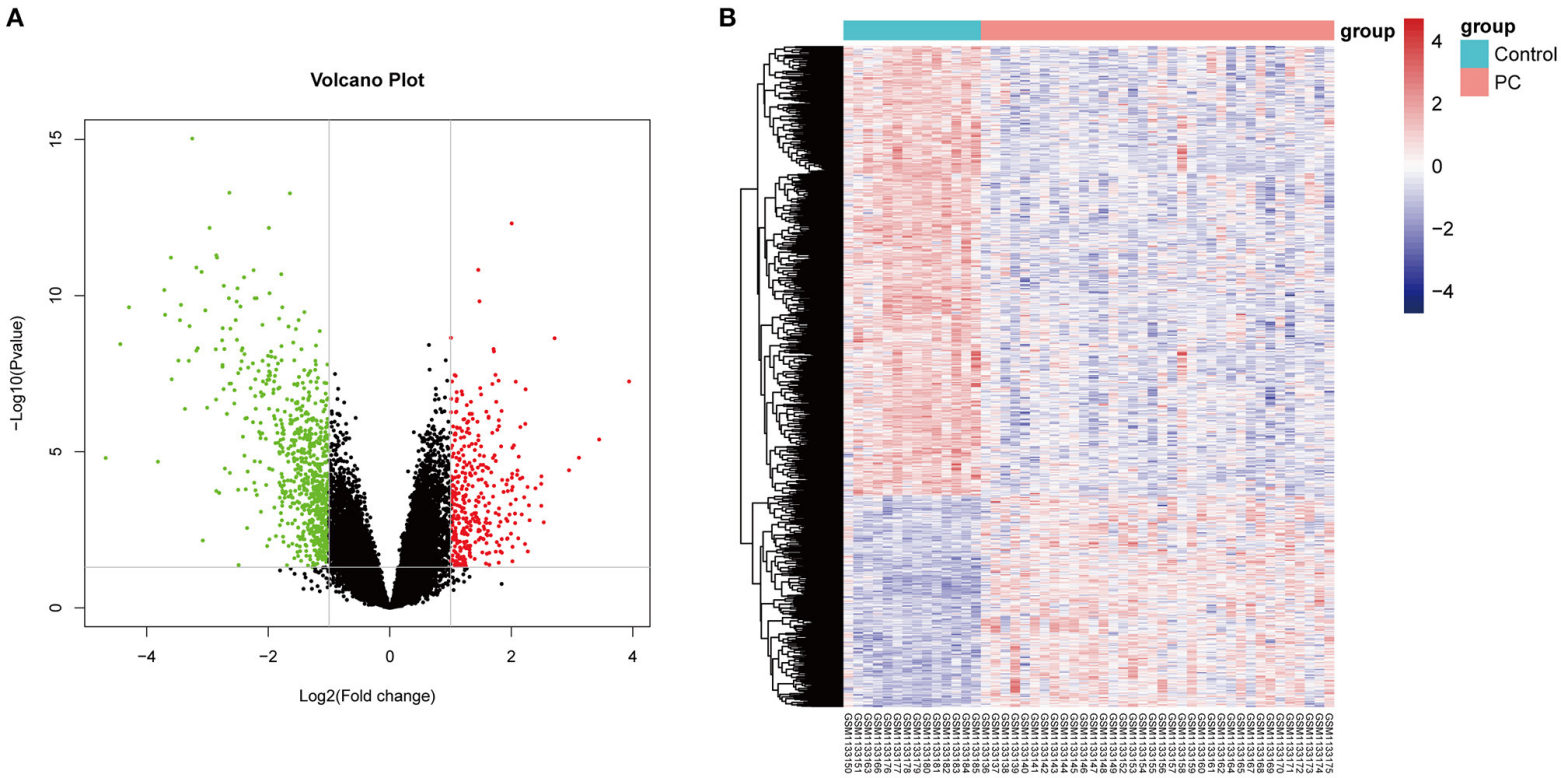
The differentially expressed genes (DEGs) in prostate cancer. **(A)** The Volcano plot shows the differentially expressed genes in the dataset GSE46602. The red color indicates the upregulated genes in prostate cancer, and the green color indicates the downregulated genes in prostate cancer. **(B)** The heat map shows the DEGs between prostate cancer tissue and normal controls.

Firstly, in order to find out the difference in genetic expression between PC tissue and normal tissue, DEGs were identified based on the RNA-Seq dataset (GSE46602) (*p* < 0.05 and log2 FC > 1.5). As seen in Figure 1A, a total of 841 DE-pcRNAs were identified, which include 269 upregulated DEGs and 572 downregulated DE-pcRNAs. What is more, to have a clearer understanding of the expression distribution of differential genes in the ischemic stroke group and the normal group, we performed heat map cluster analysis on DEGs (Figure 1B). Besides, we also screened out the TCGA database for DEGs in PCa and took the intersection with DEGs in the GEO database. A total of 385 common DEGs were obtained.

### Functional Enrichment Analysis of Common DEGs

**Figure 2:**
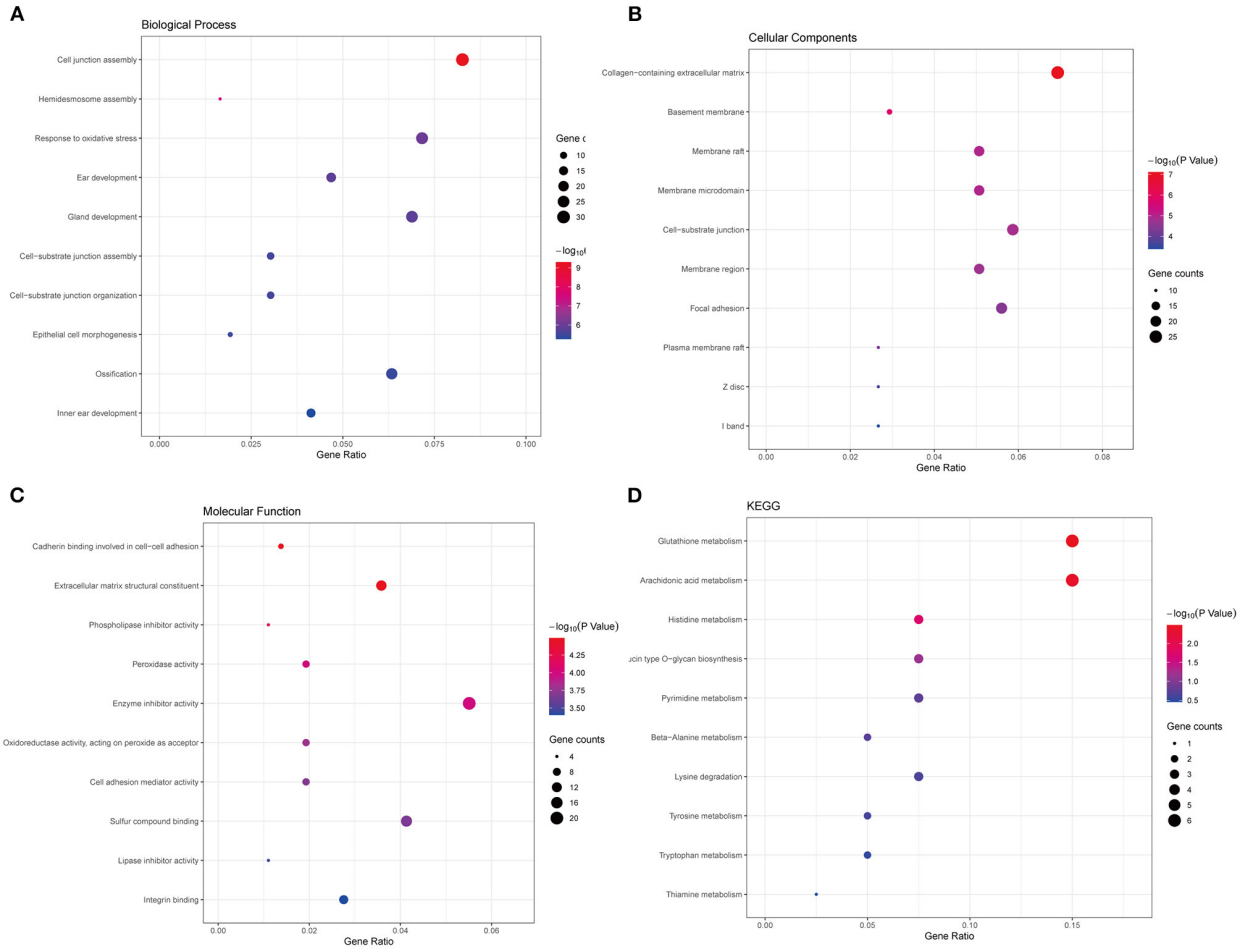
Functional enrichment analyses of the common DEGs. **(A)** The top 10 significantly enriched biological process terms. **(B)** The top 10 significantly enriched cellular component terms. **(C)** The top 10 significantly enriched molecular function terms. **(D)** The KEGG pathway analysis of the common DEGs.

Thereafter, to further uncover the function and pathways of common DEGs in PCa, functional enrichment analysis, including GO and KEGG functional enrichment analysis, was conducted. The top 10 significantly enriched terms of biological processes (BP), cell component (CC), and molecular function (MF) were shown. As seen in Figure 2A, terms like response to oxidative stress and epithelial cell morphogenesis were significantly enriched in BP (Figure 2A), collagen–containing extracellular matrix and basement membrane were significantly enriched in CC (Figure 2B), extracellular matrix structural constituent and peroxidase activity were significantly enriched in CC (Figure 2C). What is more, the results of the KEGG pathways analysis of DEGs showed that signaling pathways like glutathione metabolism, histidine metabolism, and so on were significantly enriched (Figure 2D). The functional enrichment results revealed that the common DEGs are vital to the progress of PCa.

### Cox Regression and Lasso Regression Analysis

**Figure 3:**
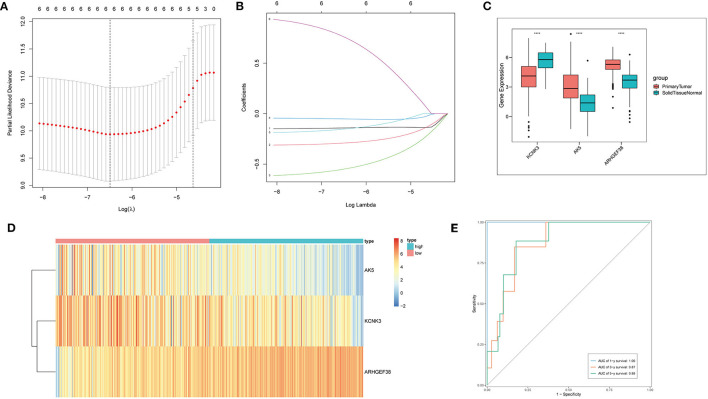
Cox regression and lasso regression analysis. **(A,B)** Cox regression and Lasso regression were performed to obtain six genes. **(C)** The relative expression of AK5, KCNK3, and ARHGEF38 in primary tumor and normal solid tissue. **(D)** The heat map of AK5, KCNK3, and ARHGEF38 expression profiles. **(E)** Time-dependent ROC curves.

In the next step, to further compress the target gene and construct immune gene models, Cox regression analysis was carried out with a threshold of *p* < 0.05. Lasso regression, a kind of compression estimation (39), was further compressed to reduce the number of genes (Figures 3A,B), and six genes were obtained. Then, multivariate regression analysis was performed. Step function was used to screen by the stepwise regression method, and finally, the risk model constructed by three genes (*KCNK3, AK5*, and *ARHGEF38*) was obtained. Next, the samples were divided into high-risk and low-risk groups based on the risk model for the following analysis. The expression level of *KCNK3, AK5*, and *ARHGEF38* in primary tumor and solid normal tissue is shown in Figure 3C. *AK5* and *ARHGEF38* have lower expression in the solid normal tissue compared to primary tumor. The expression of *KCNK3* is higher in the primary tumor. Besides, the relative expression of *KCNK3, AK5*, and *ARHGEF38* in low-risk and high-risk samples is presented in Figure 3D. The sensitivity and specificity of the risk score model were demonstrated by constructing an ROC curve. The area under the curve (AUC) was calculated to be 0.6 at 1 year, 0.87 at 3 years, and 0.88 at 5 years (Figure 3E).

### Survival Analysis of the Risk Model

**Figure 4:**
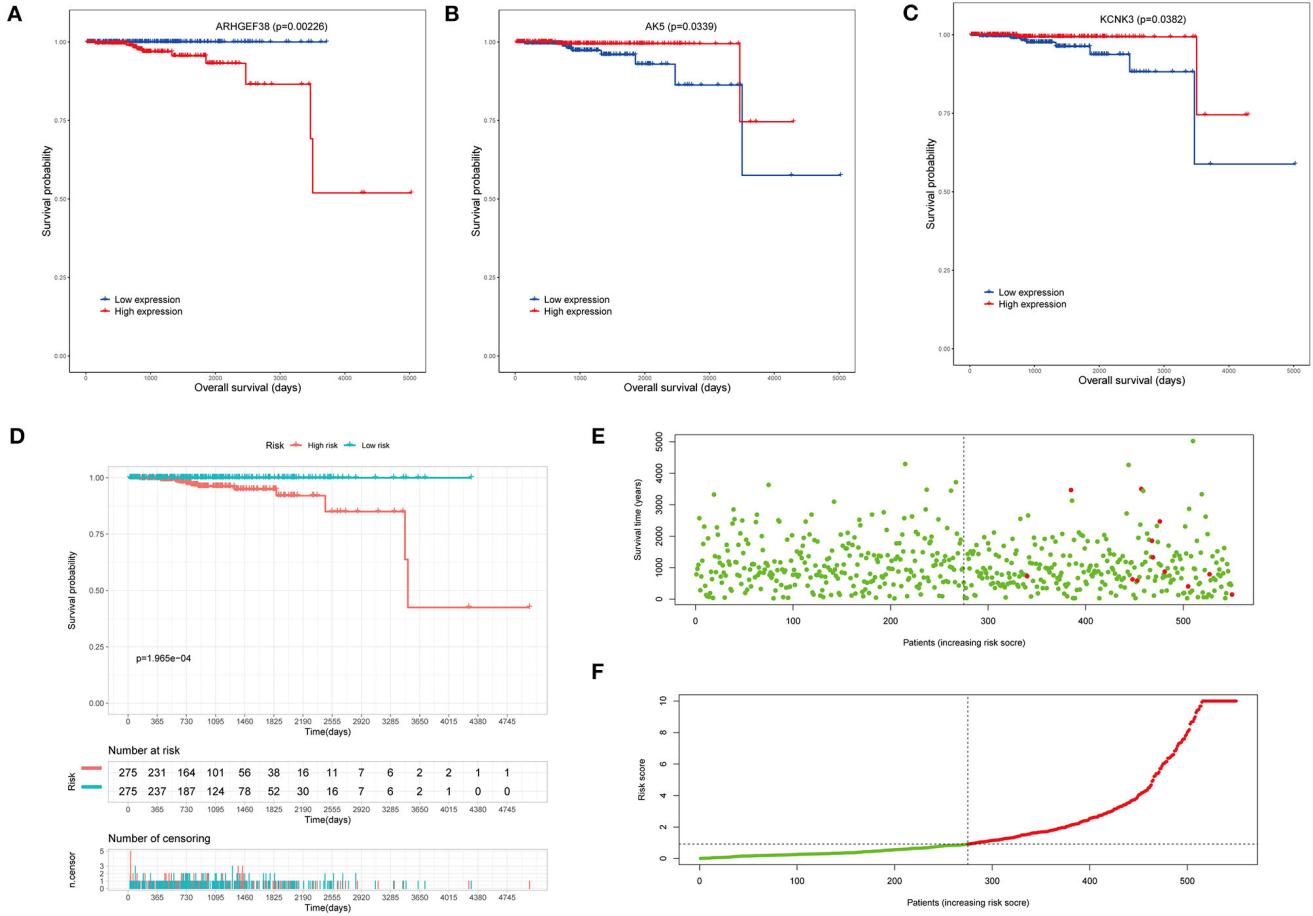
Survival analysis of the risk model. **(A–C)** The overall survival (OS) analysis in high and low *AK5, KCNK3*, and *ARHGEF38* expression samples. **(D)** Kaplan–Meier survival analyses show the OS of patients in the high-risk score and the low-risk score groups. **(E,F)** The risk plot between the high- and low-risk score groups.

To investigate the clinical significance of the risk model, the survival analyses were performed. Overall survival (OS) analysis was conducted to assess the effectiveness of the *KCNK3, AK5*, and *ARHGEF38*. As seen in Figures 4A–C, the high expression of *ARHGEF38* was associated with a lower survival probability (*p* = 0.00226), while the high expression of *KCNK3* (*p* = 0.0339) and *AK5* (*p* = 0.0382) was related to a better survival probability. The results indicated that gene changes of *KCNK3, AK5*, and *ARHGEF38* were significantly related to the OS of patients with PCa. What is more, patients with high-risk (red line) PCa presented remarkably worse OS than low-risk ones (blue line). As shown in the survival risk heat map, patients with PCa with higher risk scores had higher mortality (Figures 4E,F).

### Immune Cell Infiltration Analysis

**Figure 5:**
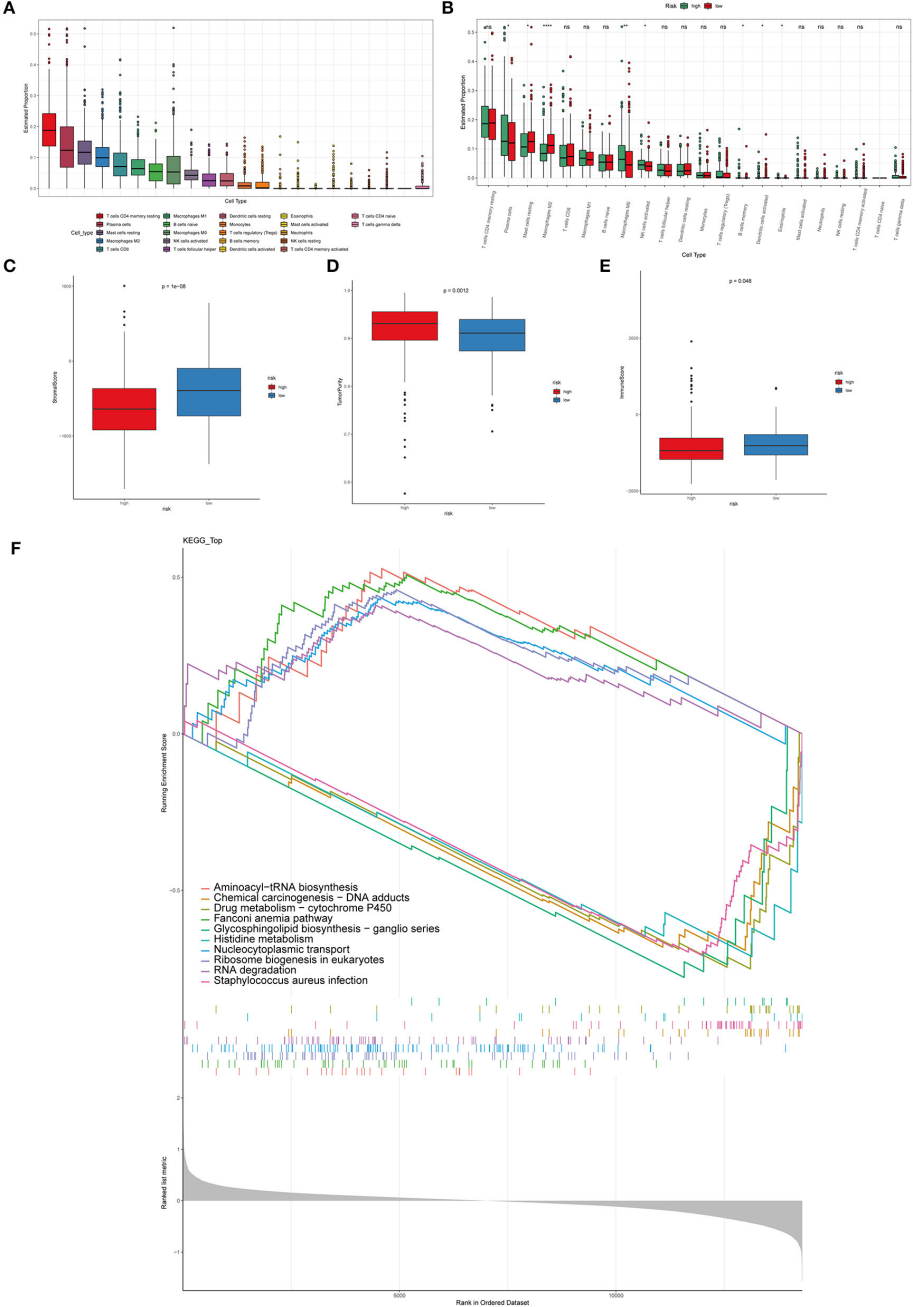
Immune cell infiltration in PCa tissues of high- and low-risk patients. **(A)** The distribution of 22 types of immune cells between primary and metastatic PCa tissues. **(B)** Comparisons between immune cells in the high- and low-risk groups in TCGA. *p < 0.05, **p < 0.01, ****p < 0.0001. The stromal score **(C)**, tumor purity **(D)**, immune score **(E)** in the high- and low-risk groups. **(F)** The GSEA analysis was performed to characterize important functional phenotypes and different gene sets between the high-risk and low-risk score groups.

The immune microenvironment is highly correlated with its overall survival (40). In order to study the correlation between immune microenvironment and the risk model, CIBERSORT algorithm, to evaluate the infiltration of twenty-two kinds of immune cells in PCa tissues, was performed (Figure 5A). Infiltration of plasma cells, mast cells resting, M0 macrophages, B cells memory, NK cells activated, M2 macrophages, dendritic cells activated, eosinophils was remarkably different in the high-risk and low-risk groups (Figure 5B). Other types of immune cells did not differ significantly between the two groups. The above results demonstrated that macrophages may be significant in the development and progression of PCa. The estimated method was utilized to predict tumor purity, stromal score, and immune. Significantly different in the high-risk and low-risk groups were shown in in Figures 5C–E. What is more, in order to distinguish the gene expression profiles between high-risk and low-risk PCa samples, GSEA analysis was performed to characterize important functional phenotypes and different gene sets between the high-risk and low-risk score groups. GSEA indicated enrichment of gene sets associated with aminoacyl-tRNA biosynthesis, chemical carcinogenesis—DNA adducts, drug metabolism-cytochrome P450, Fanconi anemia pathway, glycosphingolipid biosynthesis-ganglio series, histidine metabolism, nucleocytoplasmic transport, Ribosome biogenesis in eukaryotes, RNA degradation, staphylococcus aureus infection (Figure 5F).

## Discussion

Prostate cancer (PCa) is one of the pervasive carcinoma occurring in men and a large health burden worldwide (41). The lethality of PCa is due to the lack of treatment options that can produce a lasting response at the genetic and cellular biological levels (42, 43). The progression and pathogenic mechanisms of PCa remain unclear. Currently, analysis used for gene expression has benefit analysis for oncological research with the development in sequencing technologies. In this study, the high-throughput dataset of PCa (GSE46602) was obtained from the GEO database for further comprehensive bioinformatics analyses.

The PCa-related DEGs were screened out and explored, and the related biological processes and signaling pathways that make a better understanding of their functions were also studied. Terms like response to oxidative stress were significantly enriched in BP. Oxidative stress referred to the increase in the formation of reactive oxygen species, which destroys the body's antioxidant protection and causes a variety of diseases, including various cancers (44). Mukha et al. indicated that PCa cells can be radiosensitized by glutamine deprivation, resulting in DNA damage, oxidative stress, and epigenetic modifications (45). Glutathione-related metabolism is the main mechanism of cellular resistance to oxidative stress factors (46). The results of the KEGG analysis of DEGs demonstrated that glutathione metabolism and histidine metabolism were significantly enriched. The functional enrichment results suggest that the common DEGs were a vital regulator in the procession of PCa.

About 15% of patients with PCa are diagnosed with high-risk disease (47). Therefore, through utilizing univariate Cox and iterative lasso Cox regression analyses, a 3-gene (*KCNK3, AK5*, and *ARHGEF38*) risk signature model in PCas was constructed. The ROC curves further approved the accuracy of our risk model. It is reported that *KCNK3* influenced physiological processes, ranging from vascular tone to metabolic diet through inflammation (48). Also, *KCNK3* was correlated with prolonged survival after surgery in colorectal cancer (49). *AK5* was reported as a new prognosis marker that promotes autophagy and proliferation in human gastric cancer (33). Interestingly, *ARHGEF3* was proved to be an oncogene and may be a novel biomarker for predicting invasive PCa (50).

The 3-gene risk signature model emerges clinical significance. The results indicated that gene changes of *KCNK3, AK5*, and *ARHGEF38* were remarkedly associated with the overall survival of patients with PCa. What is more, patients with high-risk PCa have remarkably worse OS than low-risk ones. Dysregulated expression of *ARHGEF38* is associated with poor prognosis in nasopharyngeal carcinoma (51). Currently, immunotherapy has not been utilized in advanced PCa, and more novel methods are needed to overcome immune rejection and suppressive tumor microenvironment (52). As for the immune microenvironment, 8 kinds of immune related cells were remarkably various between the high-risk and low-risk score groups, including plasma cells, mast cells resting, M0 macrophages, M2 macrophages, NK cells activated, B cells memory, dendritic cells activated, and eosinophils. The risk model could further illuminate the immune-related pathogenesis of the therapeutic method by permitting early diagnosis and prognosis of PCa.

## Conclusion

In this study, we unraveled the DEGs in PCa from GEO datasets, which were further verified by TCGA data and identified the common DEGs. The functional enrichment results suggest that the common DEGs play an important role in the progress of PCa. A three-gene signature model (*KCNK3, AK5*, and *ARHGEF38*) was constructed, and the model was significantly related to cancer-related pathways, overall survival, and TME cells in PCa. This new risk model might benefit the further elucidation about the immune-related progression in PCa.

## Data Availability Statement

The original contributions presented in the study are included in the article/supplementary material, further inquiries can be directed to the corresponding author.

## Author Contributions

XL and CD designed the research. YW and LP carried out the analyses. XT performed visualization. XT and CD wrote the manuscript. All the authors reviewed and approved the final manuscript.

## Funding

This project was supported by the Youth Science and Technology Project of Hebei Provincial Health Commission (No. 20201098).

## Conflict of Interest

The authors declare that the research was conducted in the absence of any commercial or financial relationships that could be construed as a potential conflict of interest.

## Publisher's Note

All claims expressed in this article are solely those of the authors and do not necessarily represent those of their affiliated organizations, or those of the publisher, the editors and the reviewers. Any product that may be evaluated in this article, or claim that may be made by its manufacturer, is not guaranteed or endorsed by the publisher.
